# Paeoniflorin Inhibits Pulmonary Artery Smooth Muscle Cells Proliferation via Upregulating A_2B_ Adenosine Receptor in Rat

**DOI:** 10.1371/journal.pone.0069141

**Published:** 2013-07-30

**Authors:** Guoqing Qian, Jin Cao, Chan Chen, Liangxing Wang, Xiaoying Huang, Cheng Ding, Xueding Cai, Fengying Yin, Jinguo Chu, Guoxiang Li, Jinyan Ye

**Affiliations:** 1 Department of Respiratory Medicine, The First Affiliated Hospital of Wenzhou Medical College, Wenzhou, Zhejiang, China; 2 Department of Respiratory & Infectious Diseases, The Affiliated Ningbo No.1 Hospital, School of Medicine, Ningbo University, Ningbo, Zhejiang, China; 3 Department of Ophthalmology, The Affiliated Hospital, School of Medicine, Ningbo University, Ningbo, Zhejiang, China; 4 Department of Geriatric Medicine, The First Affiliated Hospital of Wenzhou Medical College, Wenzhou, Zhejiang, China; 5 Department of Medical Ultrasound, The Affiliated Ningbo No.1 Hospital, School of Medicine, Ningbo University, Ningbo, Zhejiang, China; University of Padua, Italy

## Abstract

Paeoniflorin (PF), which is the main active ingredient in the root of *Paeonia Radix*, has many pharmacological effects. Here, we investigated the effect of PF on rat pulmonary artery smooth muscle cells (PASMCs) under hypoxic conditions and explored the mechanisms of the effects. The anti-proliferative effect of PF increased in a dose dependent manner. At the highest dose (20 μmol/L), the anti-proliferative effect of PF peaked at 24 h after administration. However, the selective A_2B_ adenosine receptor (A_2B_AR) antagonist MRS1754 abolished it. PF increased A_2B_AR mRNA levels from 0.0763±0.0067 of β-actin mRNA levels (hypoxia group) to 0.1190±0.0139 (P<0.05) measured by Real Time Reverse Transcription-Polymerase Chain Reaction. A_2B_AR protein expression measured by Western Blot was also increased. PF inhibited the proliferation of PASMCs by blocking cell cycle progression in the S phase. These data indicated that activation of A_2B_AR might be involved in the anti-proliferative effect of PF on PASMCs under hypoxic conditions. The results suggested that a new mechanism of PF could be relevant to the management of clinical hypoxic pulmonary hypertension.

## Introduction

Pulmonary arterial hypertension (PAH), defined as a mean pulmonary artery pressure (PAPm) ≥25 mmHg with a pulmonary capillary wedge pressure ≤15 mmHg measured by cardiac catheterization [1]. PAH contributes to the morbidity and mortality of patients with various diseases [2]. The pathogenesis of PAH is complex and poorly understood, but chronic hypoxia is suspected as a cause of the structural changes in pulmonary arteries which might be a factor in the pathogenesis of PAH. Recent research reported that pulmonary vascular remodeling plays a key role in pulmonary arterial hypertension, which is partly due to the proliferation of pulmonary artery smooth muscle cells (PASMCs).

Adenosine receptors, which are extracellular G protein-coupled receptors, namely, A_1_, A_2A_, A_2B_, A_3_, mediate adenosine actions [3]. As A_2B_ adenosine receptor (A_2B_AR) have a lower affinity compared to other subtypes, they require micromolar concentrations of adenosine for stimulation [4]. Such high levels of extracellular adenosine are generated or released from cell under stress like hypoxia, ischemia, inflammation, and injury [5], [6]. Adenosine signaling through A_2B_AR has been shown to inhibit smooth muscle cell proliferation [7], [8], and prevent additional injury of cardiac tissues post-infarction [9].

Paeoniflorin (PF), the principal bioactive component of Paeoniae Radix [10], has been reported to have many pharmacological effects such as decreasing pulmonary artery pressure [11], relaxing vascular smooth muscle [12], analgesic [13], anti-inflammatory and anti-allergic [14], and cognition-enhancing effects [15] and the ability to activate A_1_ and A_2A_ adenosine receptors [10], [16], [17]. Whether PF has an effect on A_2B_AR in PASMCs was unknown. Accelerated proliferation of PASMCs plays a critical role in the progression of PAH. Therefore, finding new inhibitors of PASMC proliferation is an important strategy in the identification of new therapies for PAH. The goal of this study was to investigate the effects of PF on rat PASMCs under hypoxic conditions and their possible mechanisms.

## Materials and Methods

### Materials

**Figure 1:**
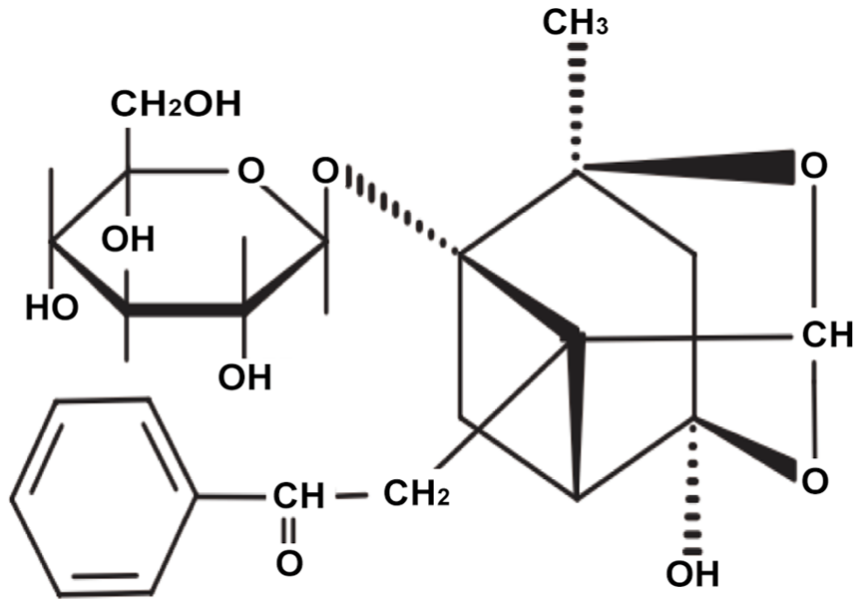
Chemical structure of paeoniflorin.

PF (Figure 1) was purchased from Silida Technology Ltd. (Tianjing, China); with a purity of ≥98.5% as determined by HPLC. PF dissolved in 50 ml sterilized saline water to prepare for stock solution with 200 μmol/L; Dilution was obtained by adding saline, and the dose was used variedly according to different administrations. Fetal bovine serums (FBS), penicillin G, streptomycin, Dulbecco's Modified Eagle Medium (DMEM, high glucose) were obtained from Gibco BRL (Gaithersburg, MD, USA). DPCPX, MRS1754, SCH58261 and IB-MECA, respectively, the selective antagonist for A_1_, A_2A_, A_2B_, A_3_ adenosine receptor and Dimethylsulphoxide (DMSO), ribonuclease (RNase), propidium iodide (PI), collagenase type I were all obtained from Sigma Chemical (St Louis, MO, USA). Cell counting kit-8 (CCK-8) was obtained from Dojindo Laboratories (Kumamoto, Japan). Cell cycle staining kit was purchased from Multiscience Biotech Co., Ltd. (Shanghai, China). Trizol was obtained from Invitrogen (Carlsbad, CA, USA). TaqMan polymerase was purchased from Promega (Madison, Wis, USA). SYBR Green Real-Time PCR was obtained from Toyobo (Osaka, Japan). A_2B_AR polyclone antibody was from Santa cruz Biotechnology, Inc (Santa cruz, CA, USA).

### Cell culture

Male Sprague-Dawley adult rats (obtained from the laboratory animal center of Wenzhou Medical College, Wenzhou, Zhejiang, China) weighing 180–220 g were used throughout the study. All the animal procedures in the present study carried out in accordance with the guidelines of Wenzhou Medical College and National Institutes of Health standards of animal care. Our study was approved by the Animal Ethics Committee of Wenzhou Medical College including permit number SCXK (Zhejiang 22–07168). The pulmonary artery from male Sprague–Dawley rats was removed under sterile conditions and washed in D-Hank's solution. The outer and inner membranes were removed under anatomy microscope. Minced arteries were digested by 0.2% collagenase type I.,then incubated in 37°C for 1–2 h. The digested pulmonary arteries were centrifuged at 1000 rpm for 7 min and then suspended by DMEM containing 20% FBS. After being dispersed, they were placed in 60 mm culture dish to incubate in incubator. The medium was changed after 72 h. Cells were maintained in the medium until 80%–90% confluence and they were subcultured with trypsin (0.25%)-EDTA (0.02%). Cells were used at passages 4–10 for experiments. The change of cells morphology was not noted and cells were identified positive of Θ -smooth muscle actin by immunofluorescence. Cells were made quiescent by incubation in serum-free DMEM for 24 h for all experiments. Hypoxia cells were performed in a CO_2_-N_2_ incubator (Heraeus, Germany) at 5% O_2_, 5% CO_2_, and 90% N_2_, 37°C. PASMCs were divided into 9 groups: (1) Normal (N), in which cells were cultured in serum-free DMEM under normoxia (21% O_2_, 5% CO_2_) for 24 h; (2) Normoxia + paeoniflorin (N+PF20), in which cells were cultured in serum-free DMEM with paeoniflorin (20 μmol/L) for 24 h under normoxia conditions; (3) Hypoxia (H), in which cells were cultured in serum-free DMEM under hypoxia (5% O_2_, 5% CO_2_) for 24 h; (4) Hypoxia +0.02 μmol/L paeoniflorin (H+PF0.02), in which cells were cultured in serum-free DMEM with paeoniflorin (0.02 μmol/L) for 24 h under hypoxia; (5) Hypoxia +0.2 μmol/L paeoniflorin (H+PF0.2); (6) Hypoxia +2 μmol/L paeoniflorin (H+PF2); (7) Hypoxia +20 μmol/L paeoniflorin (H+PF20); (8) Hypoxia + MRS1754 (H+MRS), in which cells were cultured in serum-free DMEM with MRS1754 (a selective antagonist for A_2B_ adenosine receptor, 20 nmol/L) for 24 h under hypoxia; (9) Hypoxia +20 μmol/L paeoniflorin + MRS1754 (H+PF20+MRS), in which cells were cultured in serum-free DMEM with paeoniflorin (20 μmol/L) and MRS1754 (20 nmol/L) for 24 h under hypoxia.

### Cell proliferation assay

Cell anti-proliferation by PF was detected by the CCK-8 assay. Briefly, PASMCs cultured in 96-well plates (1×104 cells/well) in the complete medium (5% CO_2_, 37°C, 95% humidity) for 24 h and then put into hypoxia incubator as described above (6 wells in each group). CCK-8 solution (10 μl/well) was added to each well. After incubate for 1 h at 37°C, the absorbance of each well was determined using a microplate reader (ELX800, BioTek Instruments, Winooski, VT, USA).

### Flow cytometry

To determine cell cycle distribution, 5×10^5^ cells were plated in 60 mm dishes and treated without or with PF at concentrations (20 μmol/L) for 24 h. Then, the cells were digested by trypsinization, after fixed in 75% ethanol, the cells were washed with phosphate buffered saline (PBS), and aliquots of the cells were resuspended in 1 ml of PBS containing 1 mg/ml of RNase and 50 μg/ml of propidium iodide (PI). After put in a dark place for 30 min at 25°C, the cell cycle was analyzed using flow cytometer (Becton, Dickinson and Company, NY, USA).

### Quantitative Real Time Polymerase Chain Reaction

We used the Trizol extract total RNA from PASMCs. RNA concentration was quantified using absorbance at 260 nm and the ratio of the absorbance at 260 and 280 nm (A260/280) was used to assess the purity of RNA. Reverse Transcription was performed using the Bio-Rad PCR system S1000 (Bio-Rad, Hercules, CA, USA) with 2 μl cDNA in a reaction volume of 8 μl containing 1 μl 10×PCR buffer and 0.8 μl primer mix. The final reverse transcription product was adjusted to 8 μl with RNase-free water. The specific primers for adenosine receptors and GAPDH: A_1_ (forward: 5′GTGATTTGGGCTGTGAAGGT3′; reverse: 5′AGGTGTGGAAGTAGGTCTGTGG3′), A_2A_ (forward: 5′CGGGAACTCCACGAAGACC3′; reverse: 5′AGCAAAGAGCCCGACGATG3′), A_2B_ (forward: 5′TCTTCCTCGCCTGCTTCGT3′; reverse: 5′GGAGTCAGTCCAATGCCAAA3′), A_3_ (forward: 5′GAAAGCCAACAATACCACGAC3′; reverse: 5′AGTGCTAGGGAGACGATGAAAT3′), and GAPDH (forward: 5′GGAAAGCTGTGGCGTGAT3′; reverse: 5′AAGGTGGAAGAATGGGAGTT3′) were designed by Primer 5.0. PCR was performed with the 7500 Real-Time PCR instrument (Applied Biosystems, Carlsbad, CA, USA). All the reactions were performed in triplicate and normalized using GAPDH as control gene. All data of the Real-Time PCR was analyzed using the Applied Biosystems 7500 System SDS Software using the standard curve method. For data analyses, the 2^−ΔΔCT^ values were determined.

### Western Blot analysis

Western Blot was used to detect A_2B_ adenosine receptor. Briefly, PASMCs were harvested, pelleted, and resuspended in ice cold lysis buffer. The Bradford protein assay was used to measure the concentration of protein. SDS-polyacrylamide gel electrophoresis (SDS-PAGE) and transferred onto a polyvinylidene fluoride (PVDF) membrane (Millipore, Billerica, MA, USA). The free protein binding sites were blocked by incubating with PBS containing 5% skimmed milk at 4°C overnight. The blot was then washed five times before incubation with goat anti-rabbit IgG in the blocking solution for 1 h. Wash the membrane again as before. Then the protein bands for other antigen-antibody complex were detected by ECL plus detection system. After scanning the X-ray film, the optical density of the immunoblots was calculated with the Quantity one–4.6.2 software (Bio-Rad, Hercules, CA, USA). A membrane incubated with a HRP-conjugated polyclonal anti-β-actin served as the control.

### Statistics

All data were shown as the mean ± the standard derivation (SD). Statistical analysis of the results were counted by one-way ANOVA followed by a Student-Newman-Keuls test. A *P*<0.05 was considered statistically significant.

## Results

### The morphology and identify of PASMCs

**Figure 2:**
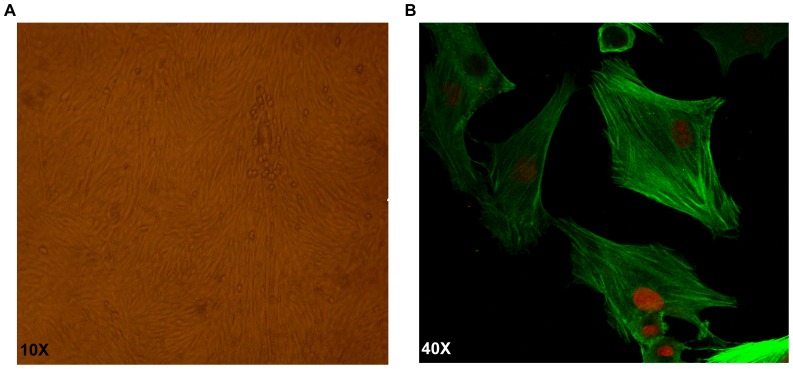
PASMCs morphology . A) Typical “hill and valley” appearance of PASMCs under phase contrast microscope. B) Immunofluorescent identification of α-smooth muscle actin was positive.

As shown in Figure 2A and Figure 2B, confluent PASMCs manifested typical “hill and valley” features under a phase-contrast microscope, and were positive for α-smooth muscle actin by Immunofluorescent staining.

### Effect of hypoxia and PF on proliferation of PASMCs

**Figure 3:**
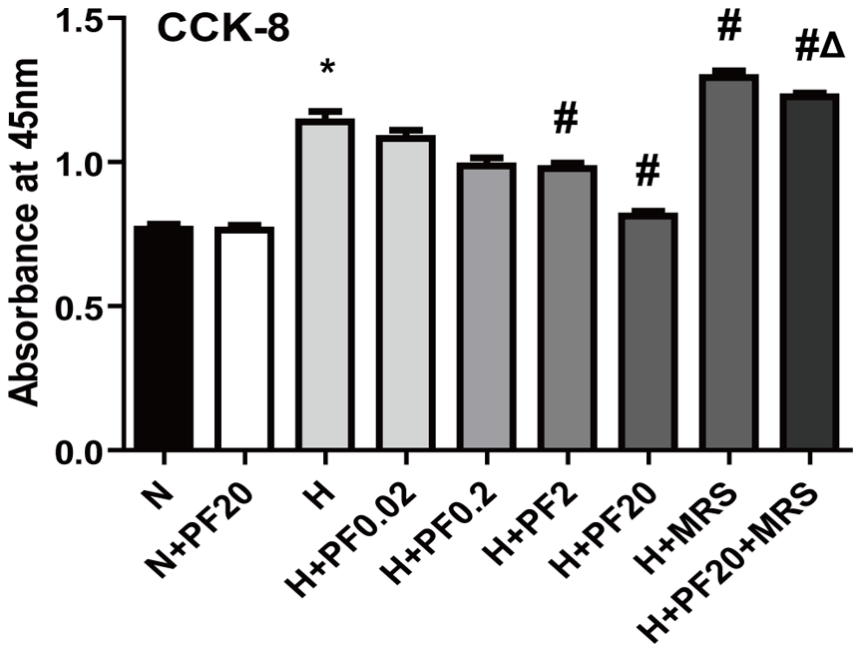
Cell viability was tested with CCK-8 . * P<0.05 vs. the N group; # P<0.05 vs. the H group; Δ P<0.05 vs. the H+PF20 group. Mean ± SD, n = 6.

The absorbance of PASMCs, which indicated the cell number, increased under hypoxic conditions when cells were cultured in complete medium with 10% FBS (Figure 3). PF suppressed the proliferation of PASMCs in a dose-dependent manner between 0.02 μmol/L and 20 μmol/L. At a PF concentration of 20 μmol/L, the proliferation of PASMCs was significantly suppressed (P<0.05 versus hypoxic group, P>0.05 versus normoxic group, n = 6). The anti-proliferative effect of PF was abolished by the selective A_2B_AR antagonist MRS1754 (20 nmol/L).

### Cell cycle analysis by Flow Cytometry

**Figure 4:**
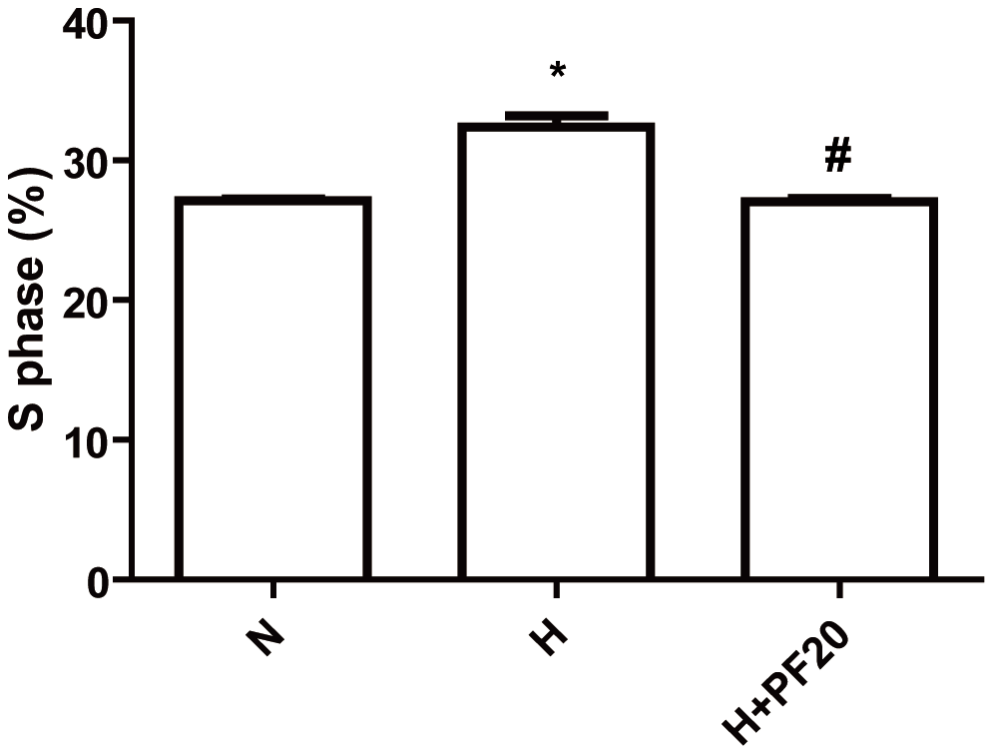
Paeoniflorin decreased the proportion of S phase in hypoxic PASMCs . PASMCs were incubated under normoxic (N), hypoxic (H) condition, or pretreated with PF (20 μmol/l) when under hypoxic condition. * P<0.05 vs. the N group; # P<0.05 vs. the H group; n = 3.

The effects of PF on cell cycle progression of PASMCs are shown in Figure 4. Compared with the normoxic group, the proportion of cells in the S phase cells was significantly increased from 27.10±0.15 % to 32.37±0.80 % in the hypoxic population (P<0.05). After treatment with 20 μmol/L PF under hypoxic conditions, the proportion cells in the S phase decreased to 27.06±0.24 %, indistinguishable from the normoxic group.

### Effects of hypoxia and PF on the expression of adenosine receptors

**Figure 5:**
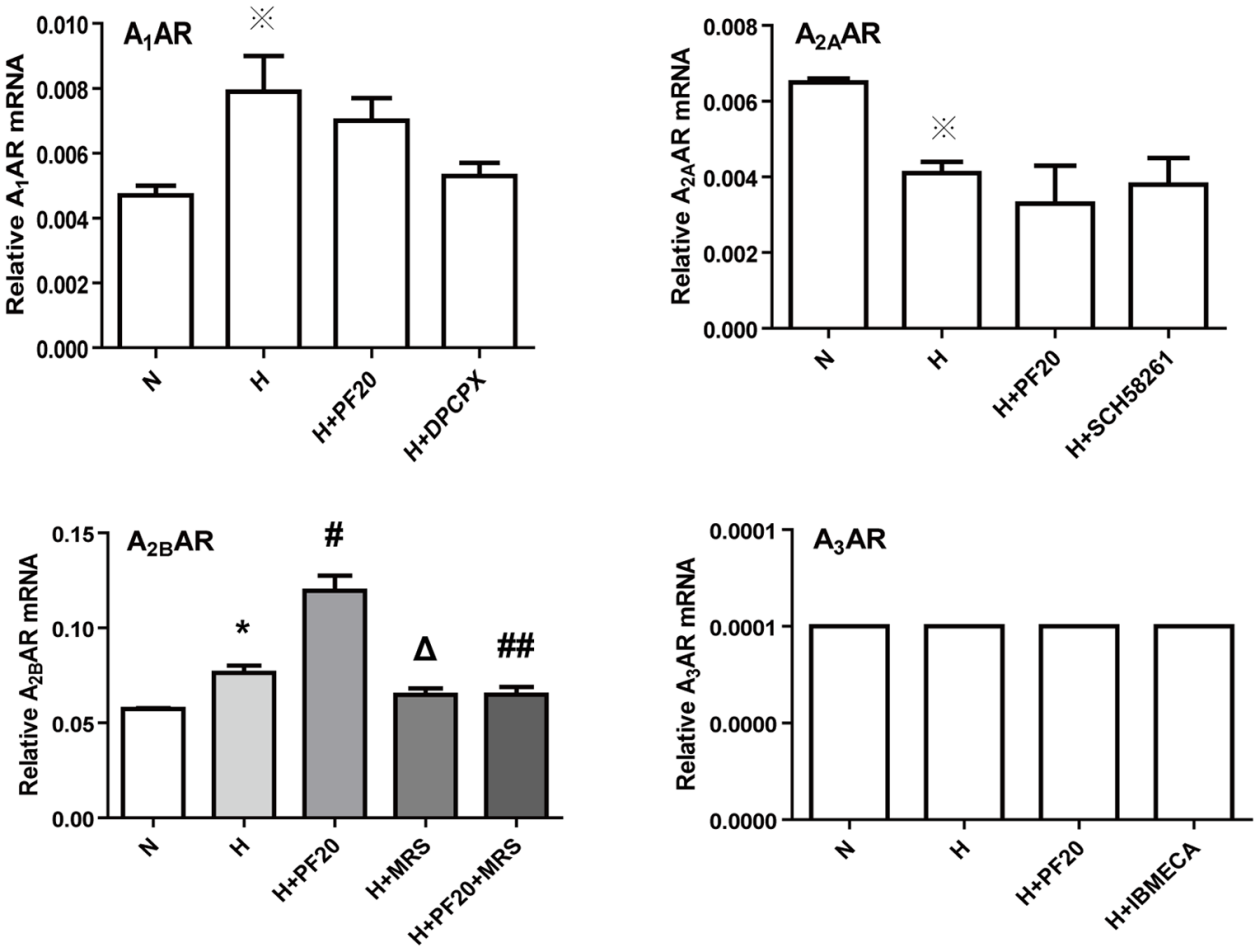
Effects of hypoxia and paeoniflorin on the expression of adenosine receptors. The PASMCs were incubated under normoxic (N), hypoxic (H) condition, or pretreated with PF (20 μmol/l) when under hypoxic condition. ※ P>0.05 vs. the N group; * P<0.05 vs. the N group; # P<0.05 vs. the H group; ## P<0.05 vs. the H+PF20 group; Δ P<0.05 vs. the H group n = 3.

**Figure 6:**
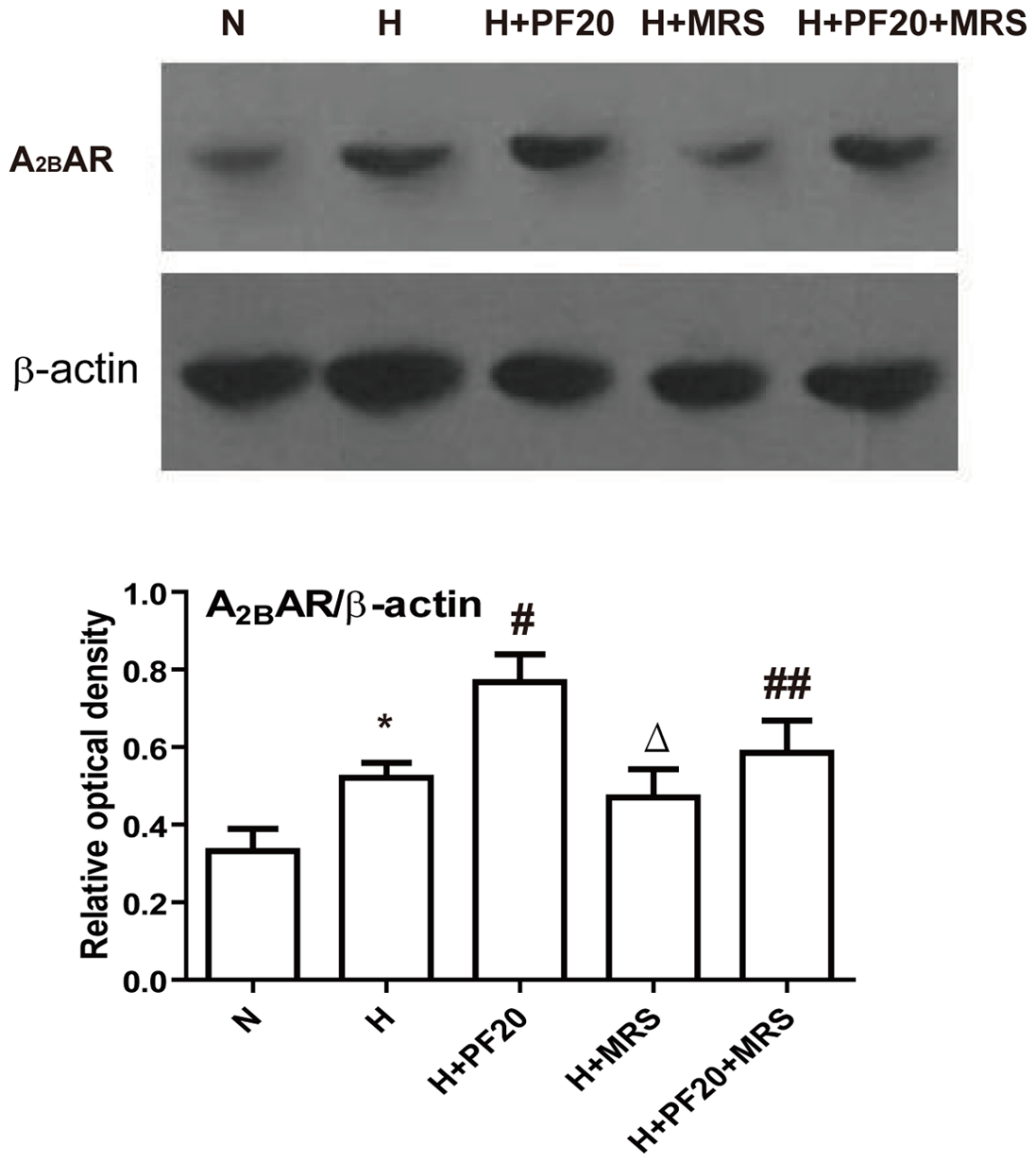
Paeoniflorin upregulate A_2B_AR protein expression in hypoxic PASMCs. Treat PASMCs were assayed for A_2B_AR protein. * P<0.05 vs. the N group; # P<0.05 vs. the H group; ## P<0.05 vs. the H+PF20 group; Δ P<0.05 vs. the H group. n = 3.

Quantitative Real-time PCR was used to measure the mRNA levels of the different adenosine receptors in PASMCs. As shown in Figure 5C, A_2B_AR had the highest expression levels among the four subtypes of adenosine receptor under normoxic conditions. A_1_AR, A_2A_AR and A_3_AR were also detected. The expression of A_3_AR was the lowest (Figure 5A, B, D). The expression levels of adenosine receptors ranked in the following order A_2B_AR > A_2A_AR > A_1_AR > A_3_AR. Under hypoxic conditions too, A_2B_AR was the predominant adenosine receptor subtype expressed in PASMCs (Figure 5C). The mRNA levels of A_2B_AR were increased from 0.0573±0.0008 to 0.0763±0.0067 (P<0.05), but the levels of A_1_AR, A_2A_AR or A_3_AR showed little change. After pretreatment with PF under hypoxic conditions, the expression levels of A_2B_AR increased significantly to 0.1190±0.0139 (P<0.05, versus to hypoxic control group). As shown in Figure 6, the expression of A_2B_AR protein was detected by Western Blot. A_2B_AR protein expression increased in the hypoxic control group compared to the normoxic control group. PF induced a further increase in the A_2B_AR protein levels in hypoxic rat PASMCs. However, the effects of PF, which increased the expression of A_2B_AR mRNA and protein under hypoxic conditions, were abolished by MRS1754 (Figure 5C, Figure 6).

## Discussion and Conclusions

To our knowledge, this is the first report about the effects of PF and hypoxia on rat PASMCs, and a novel mechanism is proposed for the role of PF and A_2B_AR in hypoxic pulmonary hypertension. PF caused inhibition of pulmonary artery smooth muscle cells growth by activating A_2B_AR and inducing its expression.

Several reports demonstrated the presence of adenosine receptors in lung in different species. A_1_AR, A_2B_AR, and A_3_AR were detected in rat airway smooth muscle cells [18]. It was reported that the levels of A_2B_AR transcripts were the highest in human bronchial smooth muscle cells, A_1_AR and A_2A_AR transcripts were detected also, but A_3_AR transcripts were below detection limit [19]. Similarly, our study also shows that among the four subtypes of adenosine receptors in rat PASMCs, the expression of A_2B_AR was highest with lower levels of A_1_AR and A_2A_AR. However, A_3_AR mRNA was also detected at a lower level. The differences of adenosine receptors between rat and human are not entirely surprising because it is possible that the effects of hypoxia on the expression of adenosine receptors are cell- and tissue-specific [20], [21].

Hypoxia is common in a variety of disease states, like hypoxic pulmonary hypertension, tumor growth, and ischemia etc. Cell hypoxia is a potent stimulus for adenosine release. One study demonstrated that hypoxia downregulated high-affinity A_2A_AR and upregulated low-affinity A_2B_AR in Human Umbilical Vein Endothelial Cells [21]. In other reports A_2B_ adenosine receptors were upregulated under hypoxic conditions [22]–[24]. Here, we demonstrated that the expression of certain adenosine receptor subtypes in rat PASMCs changed greatly under hypoxic conditions. Hypoxia upregulated the expression of low-affinity A_2B_AR, but it did not change the expressions of A_1_AR, A_2A_AR, and A_3_AR.

Earlier reports showed that A_2B_AR is a tissue protector and that it inhibited Vascular Smooth Muscle Cell proliferation [8], [25], [26]. However, whether the activation of A_2B_AR in PASMCs could inhibit PASMCs proliferation was unknown. The key finding of our study is that A_2B_AR can inhibit the proliferation of PASMCs under hypoxic conditions.

PF is the main active ingredient in the root of *Paeonia Radix*, as Chinese herbal medicine. It has been reported to have many pharmacological effects. Many researchers focused on the effects of paeoniflorin on lung [11], brain [27], or plasma [28] etc. It was reported that PF could inhibit the proliferation in pig PASMCs [11], however, the mechanism of this paeoniflorin effect was not clear. Liu *et al* reported the activation of A_1_AR might be involved in PF-induced neuroprotection in cerebral ischemia in rats, but this binding manner was different from that of classical A_1_ adenosine receptor agonists [10]. PF increased erythropoietin mRNA levels by activating PI-3 kinase pathway through A_1_ and A_2A_ adenosine receptors under hypoxic conditions [29]. Previously, it was unknown whether PF can upregulate A_2B_AR under hypoxic conditions. Here we showed that pretreatment of PASMCs with under hypoxic conditions significantly upregulated the expression of A_2B_AR, while it did not change the expression of A_1_, A_2A_, and A_3_ adenosine receptors. To further investigate the possible mechanism underlying the effect of PF on A_2B_AR, the PASMCs were pretreated with MRS1754, a selective A_2B_AR antagonist which abolished PF –induced upregulation of A_2B_ adenosine receptors. To further clarify the detailed mechanism, more studies are required.

In conclusion, PF is effective in inhibiting the proliferation of PASMCs via upregulating the expression of A_2B_ adenosine receptors and by activating it. These findings might provide a new theoretical basis for the use of PF in the management of clinical hypoxic pulmonary hypertension.
